# Development and characterization of sustainable chitosan film enriched with ashwagandha extract as an alternative packaging material for enhancing shelf life of fresh-cut fruits

**DOI:** 10.1039/d5ra01102g

**Published:** 2025-04-17

**Authors:** Mohamed Gouda, Nashi K. Alqahtani, Manal F. Abou Taleb, Ibtisam Alali, Hany M. Abd El-Lateef

**Affiliations:** a Department of Chemistry, College of Science, King Faisal University Al-Ahsa 31982 Saudi Arabia mgoudaam@kfu.edu.sa hmahmed@kfu.edu.sa; b Date Palm Research Center of Excellence, King Faisal University Al-Ahsa 31982 Saudi Arabia; c Department of Food and Nutrition Sciences, College of Agricultural and Food Sciences, King Faisal University Al-Ahsa 31982 Saudi Arabia; d Department of Chemistry, College of Science and Humanities, Prince Sattam Bin Abdulaziz University Al-Kharj 11942 Saudi Arabia; e Department of Chemistry, College of Science, Jouf University Sakaka Aljouf 72341 Saudi Arabia

## Abstract

The current study aimed to develop biodegradable chitosan (Cs) films enriched with Ashwagandha (ASH) extract as an active packaging material to extend the shelf life of fresh-cut strawberries. The ASH extract, obtained through methanolic extraction, demonstrated significant antimicrobial and antioxidant activities, as confirmed by Gas Chromatography-Mass Spectrometry (GC-MS), which identified 12 bioactive compounds, including *n*-hexadecanoic acid (30.42%) and *cis*-13-octadecenoic acid (31.68%). The ASH loaded Cs films, prepared at varying concentrations of ASH extract, were characterized for surface morphology, water vapor transmission rate (WVTR), oxygen permeability (OP), and water contact angle (WCA). The films' hydrophilicity was improved with increasing ASH concentration, reducing the WCA from 112.4° (Cs) to 77.3° (ASH6/Cs). Antibacterial evaluation of the ASH3/Cs film revealed potent inhibition against *Salmonella typhi* (35.49 mm), *Pseudomonas aeruginosa* (34.85 mm), *Bacillus subtilis* (31.64 mm), *Listeria monocytogenes* (31.71 mm), and *Candida albicans* (29.25 mm). When tested over a 9-day storage period, the ASH3/Cs film effectively preserved fresh-cut strawberries, reducing microbial growth, weight loss, and decay compared to polyethylene (PE) packaging. These results highlight the potential of ASH3/Cs film as a sustainable and efficient alternative for food packaging, offering enhanced preservation and safety for perishable fruits.

## Abstract 2

This study aimed to develop biodegradable chitosan (Cs) films enriched with Ashwagandha (ASH) extract as an active packaging material to extend the shelf life of fresh-cut strawberries.

## Graphical abstract

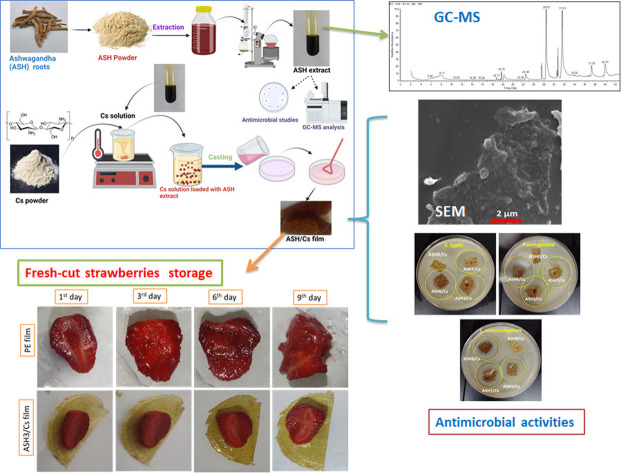

## Introduction

Strawberries, from the genus *Fragaria* in the Rosaceae family, are valued for their nutritional and health benefits, including reduced cholesterol and antioxidant activities. However, contamination risks from pathogens like *Salmonella* and Norovirus have been highlighted, emphasizing the need for effective decontamination treatments to ensure safety during handling and processing.^1^ Fresh-cut strawberries are highly perishable due to their high water content and the exposure of internal tissues to air after cutting. This exposure accelerates moisture loss, microbial contamination, and enzymatic browning, leading to a rapid decline in quality. The main challenges in preserving fresh-cut strawberries include maintaining their visual appeal, texture, flavor, and nutritional value while preventing microbial spoilage.^2^

Packaging is a material barrier separating the product from the outside environment. In addition to its four essential functions, containment, protection, convenience, and communication, packaging can also provide the proper physicochemical conditions, thereby ensuring the extension of shelf life and maintaining food products' quality and safety during transport and storage.^3^ Polymers most frequently used in packaging are petroleum-based: polypropylene (PP), several grades of polyethylene (PE), polystyrene (PS), polyvinyl chloride (PVC), and polyethylene terephthalate (PET).^4^ The use of such polymers can help reduce food loss by preserving freshness. At the same time, their increased application has made it one of the significant environmental problems in waste management.^5^

Furthermore, these materials are non-biodegradable, leading to persistent environmental pollution as they accumulate in landfills and oceans. The production and disposal of synthetic plastics contribute to greenhouse gas emissions and ecological degradation. Additionally, these materials can leach harmful chemicals into food products, posing health risks to consumers. Efforts to recycle plastics are often inadequate, with a substantial portion still ending as waste. Consequently, a growing demand for sustainable, biodegradable alternatives can mitigate these environmental and health concerns.^6^ The combustion of these polymers and the release of CO_2_ and other greenhouse gases pose a significant risk to the ozone layer and add to the peril of global warming. Such environmental issues reveal the need to create sustainable packaging materials that must be biodegradable to avoid such hazardous effects.^7^ The adoption of biopolymers, naturally degradable materials that do not generate toxic byproducts, offers the food industry a sustainable solution to mitigate the environmental impact of plastic waste in the future.^8^ The development of sustainable and biodegradable polymers derived from natural sources such as cellulose, starch, alginate, and chitosan (Cs) are being explored as alternatives.^9^ Among these, Cs is a promising biopolymer due to its excellent film-forming properties, biodegradability, biocompatibility, and inherent antimicrobial activity.^10^

Moreover, Cs, derived from the deacetylation of chitin found in the exoskeletons of crustaceans, is a natural cationic polymer formed by deacetylation of chitin, and it barely forms most of the crustacean shells. The linear backbone heteropolysaccharide comprises units of glucosamine and *N*-acetylglucosamine, which are linked through β-1,4 linkages.^11^ Owing to its benign and biodegradable nature, nontoxicity, and biocompatibility, it has attracted the interest of many researchers in diverse applications, including the biomedical, agricultural, and food packaging fields. Thus, all these properties make it predominant in usage as an environment-friendly alternative to synthetic polymers for sustainable packaging solutions.^12^ In addition, Cs films are selectively permeable to carbon dioxide and oxygen and have relatively high robustness and mechanical properties.^13^ Besides its barrier task, the Cs film may also serve as the carrying system of bioactive compounds with antioxidant or antibacterial properties.^14^ Bioactive compounds also have broad-spectrum antimicrobial properties, effectively inhibiting microorganisms such as bacteria, yeast, and fungi, which are common causes of food spoilage.^15^ Consequently, this makes Cs a valuable component in active food packaging material to restrict the growth of microorganisms and prolong the durability of consumable food products.^16^ The antibacterial activity of Cs films is improved by including bioactive compounds. Natural extracts, essential oils, are often added to enhance the mechanical properties and antimicrobial effectiveness of Cs.^17^ Such these extracts or essential oil enhance the activity of Cs and provides better protection against microbial contamination in the film, since they could provide antioxidant and/or antimicrobial activities to the packaging.^18^

Ashwagandha (*Withania somnifera*), a well-known medicinal herb in traditional Indian medicine, possesses various bioactive compounds with antimicrobial properties. Methanolic extracts of Ashwagandha (ASH) have been shown to exhibit significant antimicrobial activity, which can be harnessed to enhance the protective qualities of Cs films.^19^ Chemical analysis of various parts of ASH showed the presence of a huge number of compounds belonging to different classes of chemical entities.^20^ Most of the biologically active constituents are alkaloids, steroidal lactones that contain withanolides and withaferins, and saponins having an extra acyl group.^21^ Studies have validated that the methanolic extracts of the whole of ASH itself possess some important antibacterial activity against a mix of pathogenic bacteria, including *E. coli*, *Pseudomonas aeruginosa*, *Staphylococcus aureus*, *Streptococcus mutans*, and *Candida albicans*.^22^ Alam *et al.*^23^ evaluated the antioxidant and antibacterial activity of the 80% aqueous methanolic extract of *W. somnifera* roots (WSREt), fruits (WSFEt), and leaves (WSLEt).

In addition to its antimicrobial benefits, ASH extract has demonstrated significant antioxidant activity through its polyphenolic and flavonoid constituents. These compounds function *via* electron transfer (ET) and hydrogen atom transfer (HAT) mechanisms to scavenge free radicals and inhibit lipid peroxidation.^24^ Studies have suggested that incorporating ASH extract into bio-based polymers such as Cs enhancing the oxidative stability of food products by reducing oxidative degradation during storage. This property is particularly valuable in food packaging applications where oxidative spoilage contributes to quality deterioration and shelf-life reduction.^25^ Research has indicated that integrating ASH extract into biopolymeric matrices improves films' mechanical and barrier properties, making them more effective in controlling moisture loss and oxygen permeability.^26^

This study focuses on designing an edible Cs films incorporated with ASH extracts to prolong the shelf life of fresh-cut strawberries. The objectives include isolating and identifying the bioactive constituents in ASH extracts and examining their antibacterial and antioxidant activities. Besides, the investigation aims to design and characterize biodegradable antimicrobial Cs films before and after loading with ASH extracts to hinder microbial burden and control spoilage, thereby maintaining strawberry freshness. Eventually, the potential of these edible Cs films loaded with ASH extract to inhibit bacterial growth and their total phenolic content (TPC), total flavonoid content (TFC), and antioxidant activity are evaluated.

## Materials and methods

Chitosan (Cs, medium molecular weight, viscosity 200–800 cP, 75–85% deacetylated) was obtained from Sigma-Aldrich Co., USA. Plant material (Ashwagandha roots) was purchased from local market located in Al-Ahsa, KSA. All the solvents and reagents were obtained from Sigma-Aldrich, UK. Culture media compositions for microbial culture enrichment were obtained from HiMedia Company, India.

### Ashwagandha roots extraction method

Fresh Ashwagandha (ASH) roots were thoroughly washed with distilled water to remove impurities and then cut into small parts. The parts were spread over a sieve with a mesh size ranging from 10 to 30 mesh. Hand-picking was carried out to take out any kind of infected roots. The parts that appeared to be healthy were selected, and these were air-dried at a temperature of 60 °C overnight. After that, these roots were grinded to fine powder with the help of an electric blender. For extraction of bioactive constituents, 100 g of the resultant fine power were allowed to marcenate in a volume of 500 mL of methanol. The mixture was placed in a shaking incubator at a temperature of 40 °C for 6 h. Then, the mixture was kept in a dark and cold place for 24 h. After the completion of the extraction process, the mixture was filtered in order to take out the solvent layer containing the bioactive constituents from the residues of the plant. The crude methanolic ASH extract was evaporated to remove the solvent residues and concentrate the extract using rotary evaporator.

### Qualitative analysis of phytochemical constitutes

In the ongoing study, the ASH extract were subjected to qualitative analysis for various phytochemical compounds, including alkaloids, phenolics, flavonoids, anthraquinones, glycosides, tannins, saponins, terpenoids, and reducing sugars as per standard methods.^27^

### Identification of bioactive composition of ASH extract

The constituents of the ASH extract were analyzed using Gas Chromatography-Mass Spectrometry (GC-MS) with a QQQ 7890B instrument (Agilent, Santa Clara, USA). The system was equipped with a fused silica capillary column (30 m length, 250 μm thickness, and 0.25 μm internal diameter). Helium was used as the carrier gas at a constant flow rate of 1 mL min^−1^. The oven temperature was initially set at 40 °C and programmed to increase gradually up to 300 °C. The resulting spectra were compared against the Wiley Library database for the identification of the extract’s components.^28^

### Biological characteristics of ASH extract

#### Evaluation of antioxidant activity

The 2,2-diphenyl-1-picryl-hydrazyl (DPPH) scavenging activity of ASH extract was pefromed. One mL of ASH extract was added to a 4 mL methanol solution of DPPH (75 × 10^3^ mol L^−1^). The reaction of the solution was kept in the dark for 30 min at room temperature. The bleaching of the dark solution of DPPH 26 determined antioxidant activity.

#### Antibacterial properties

The antimicrobial activities of the ASH extract were tested against several microbes, namely *Salmonella typhi*, *Pseudomonas aeruginosa*, *Bacillus subtilis*, *Listeria monocytogenes*, and *Candida albicans*. The microbicidal activity of the above extracts was performed using agar diffusion method. The bacterial strains were cultured on the Mueller–Hinton (MH) agar, while *C. albicans* were cultured on Malt extract (ME) agar plates. The bacterial strains were inoculated at a concentration of approximately (10^6^ CFU mL^−1^). Wells of approximately 6 mm diameter were made in the agar, and 100 μL of individual extracts were pipetted into the wells. The inhibition zones around the wells were recorded in mm, from which we get the antimicrobial activities of the extract.^29^

#### Minimum inhibitory concentration (MIC) and biocidal concentration (MBC)

MICs were determined on a 96-well plate format, where different concentrations of ASH extract were prepared from 50–800 μg mL^−1^. Bacterial and fungal culture suspensions were added, and plate incubation was done at 37 °C for bacteria and 28 °C for yeast. Resazurin solution was also added to each well, and thereafter, changes in color were observed to determine the MIC. The MBC of ASH extract was estimated to ensure the killing of microbes.^30^

#### Preparation of Cs film loaded with and without ASH extract

**Fig. 1:**
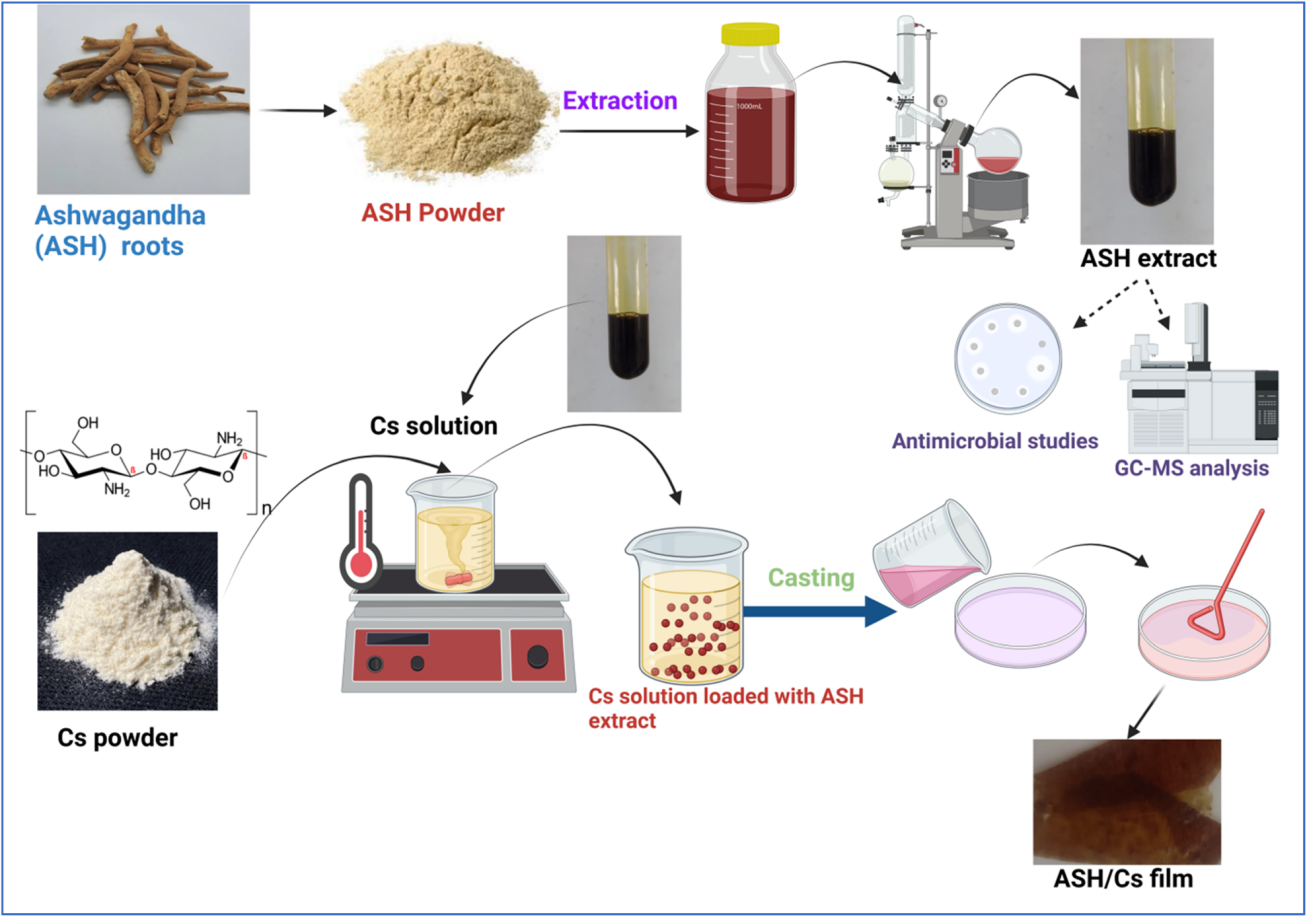
A schematic diagram illustrating the fabrication steps of Cs films before and after loading with ASH extract.

Cs solution was prepared by dissolving 6 g in 300 mL of 1% acetic acid with stirring for 12 h at room temperature. Then, Cs solution was mixed with ASH extract (concentrated extract) solution in mL as follows: 30/0, 29/1, 27/3, and 24/6. The solutions were mixed well under the effect of the ultrasonic probe for 5 min. The blended solutions were placed in an ultrasonic water bath to remove the air bubbles and directly cast them by pouring them into flat silicon-coated Petri dishes. Finally, these dishes containing mixed solutions of both Cs and the extract were left to dry in ambient conditions for 48 h. After drying, the films were carefully removed and stored in plastic bags until characterization and application. Using these different ratios of both Cs and ASH extract solutions, four film samples were prepared, coded as follows: ASH1/Cs, ASH3/Cs, and ASH6/Cs; these samples were compared with Cs film without ASH loading (ASH0/Cs). As shown in Fig. 1, the steps of fabrication of Cs films before and after loading with ASH extract were illustrated.

### Characterization

A scanning electron microscope (SEM, TESCAN, VEGA 3, Czech Republic) was employed for film examination. The film samples were fixed on the aluminum stubs with carbon tape adhesive. Quorum Techniques Ltd's sputter coater (Q150t, England) was used to coat the samples with a gold for 3 min. The molecular structure of the prepared films, ASH0/Cs, ASH1/Cs, ASH3/Cs, and ASH6/Cs, was determined by employing Attenuated Total Reflectance-Fourier Transform Infrared (ATR-FTIR) spectrometer (Vertex 70, Bruker, Germany). The spectra were collected over a range of 4000 to 400 cm^−1^ with a resolution of 4 cm^−1^. Using the sessile drop method at 25 °C, the contact angles of the films were determined using a data physics instrument (model: OCA 15EC, Germany).

The water vapor transmission rate (WVTR) of the films was determined using the ASTM method (1993) at 25 °C in a medium with a constant relative humidity of 83%. Additionally, after submerging the films in room-temperature water for 48 h, the film's water uptake (*Q*%) was evaluated following ISO62-1980(E). In compliance with the national standard of the Chinese GB1038-2000, the determination of oxygen permeability (OP) was carried out by the differential-pressure method utilizing a VAC-V1 Gas Transmission Tester (Jinan, China).

### Biological evaluation of prepared packaging materials

#### Total antioxidant capacity and phytochemical analysis

The total antioxidant activity (TAC), in conjunction with the phytochemical profile in terms of total phenolic content (TPC) and total flavonoid content (TFC), was estimated for the films (ASH1/Cs, ASH3/Cs and ASH6/Cs). The investigation was conducted to validate the release of bioactive molecules of ASH after loading in the Cs film.

The phosphomolybdate method was employed and applied to determine the total antioxidant capacity (TAC) of each film loaded with ASH extract, which is based on the determination of the reduction of Mo(vi) to Mo(v) by the antioxidant species, thereby, a green phosphate/Mo(v) complex was formed at acid pH and determined by spectrophotometry.^31^

The amount of TPC was estimated using the Folin–Ciocalteu (FC) reagent as described in the method of Pérez *et al.*^32^ The absorbance was taken at 760 nm in a spectrophotometer. The standard used was gallic acid, and the results were expressed as mg of gallic acid equivalents per gram of dry weight of ASH extract (mg GAE per g DW).

The total flavonoid content (TFC) was determined by mixing 1.2 mL of the sample with 0.1 mL of a solution of 5% AlCl_3_ (w/v) and 1.4 mL of a mixture of acetic acid and methanol in the ratio 1 : 19. The solution was kept in the dark for 30 min in the incubator. After that, the absorbance was measured at 430 nm using a spectrophotometer.^33^ The standard used was quercetin, and the results were expressed as quercetin equivalents in the concentration of mg per gram of dry weight of the extract (mg QE per g DW).

#### Inhibition zone assay for tested films

Four different materials (ASH0/Cs, ASH1/Cs, ASH3/Cs, and ASH6/Cs) were assessed for their ability to inhibit the growth of foodborne pathogens using the disc diffusion method. Before testing, samples measuring 1 cm × 1 cm were disinfected for a period of 60 min. The sterilized sample portions were placed onto the agar surfaces after spreading 300 μL of diluted bacteria onto Mueller–Hinton Agar (MHA) and Sabouraud Dextrose Agar (SDA) plates. Following an incubation period, the zones of inhibition around the samples were both measured.

#### Toxicity assessment

Comprehensive biocompatibility and toxicity experiments were performed to guarantee the safety and suitability of the ASH extract and Cs film after loading ASH extract (ASH0/Cs, ASH1/Cs, ASH3/Cs, and ASH6/Cs films) for application in human and environmental contexts. The toxicity study-specific Microtox analyzer 500 system was used. *Vibrio fischeri*, a lyophilized photobacterium, is used in this system along with reconstitution reagents and a diluent (2% NaCl). There were several stages in the testing process. The lyophilized cells of *V. fischeri* were first gradually mixed with a refrigerated reconstitution solution. For 5, 10, and 15 min, the sample and photobacteria combination were incubated at precisely controlled cooling temperatures suitable for the *V. fischeri* bacteria.

#### Evaluation of prepared films effect on fresh-cut strawberries preservation

To estimate the effect of the prepared films on the quality of fresh-cut strawberries, an experiment was done. Strawberries freshly harvested and selected to be of the same size and shape were bought from a local fruit store, and they were put in Petri dishes without any cleaning. The strawberries were then covered by the prepared films and were sealed with plastic wrap. Fresh-cut strawberries of approximately equal gloss and maturity were chosen for more systematic testing. The selected strawberries were thoroughly washed with water and air-dried at room temperature. The fresh-cut strawberries were packed in different packaging films (PE material, ASH0/Cs, ASH1/Cs, ASH3/Cs, and ASH6/Cs). All of them were preserved under controlled conditions (25 °C and 70% humidity) for 14 days. The critical physiological and chemical parameters were measured and recorded every two days to test the changes and study the preservation's efficacy. Changes indicating a hint of rotting or degradation were tested to measure the effectiveness of preservation.^34^

For the microbial growth monitoring of the bacteria and fungi, 1 g of fresh-cut strawberries of each group was homogenized with 9 mL of sterile NaCl and was blended for 5 min to make a homogenized solution. The solution was then diluted with the dilution according to the requirement. For the microbial counting, 1 mL of the diluted sample was shaken well with 25 mL of the plate count agar medium and poured directly into sterile plates. The plates were incubated at 37 °C for 24 h for bacterial counts and 28 °C for 3–4 days for fungal count. Thereafter, the total number of colonies on each plate was counted for the microbial load.^35^

Weight loss rate: the initial weight (*W*_0_) of the fresh-cut strawberries was taken after the application of coating with the films and also after the curing. The weight (*W*_i_) of the strawberries was taken at intervals of two days. The rate of weight reduction in fresh-cut strawberries was determined by employing eqn (1).

1 Weight loss (%) = (*W*_0_ − *W*_i_)/*W*_0_

### Statistical analysis

To determine the statistical significance of the effect of thyme extracts on the microorganisms under investigation, an analysis of variance (*ANOVA*) was performed utilizing Microsoft Excel. The collected data underwent analysis, and graphical depictions of the findings were produced using GraphPad software (version 6.0, Prism, San Diego, CA, USA).

## Results and discussion

### Qualitative identification of phytochemicals constitutes

The qualitative analysis of the crude methanolic ASH extract shows a diverse range of biologically active compounds that collectively contribute to its broad biological properties (Table 1). The identification of alkaloids, phenolics, flavonoids, glycosides, tannins, saponins, terpenoids, and reducing sugars underscores the potential of the extract for a variety of therapeutic uses, including antioxidant, anti-inflammatory, antimicrobial, and anticancer effects.

**Table 1:** Qualitative assays for phytochemical constituents of crude methanolic ASH extract

Items	ASH extract	Notes
Alkaloids	++	— No reaction
Phenolics	+++	+ Reaction in >30 min
Flavonoids	++	++ Reaction in 5–30 min
Anthraquinones	—	+++ Rreaction within 5 min
Glycosides	++	
Tannins	++	
Saponins	+++	
Terpenoids	+++	
Reducing sugars	+++	

### GC-MS analysis of ASH extract

**Fig. 2:**
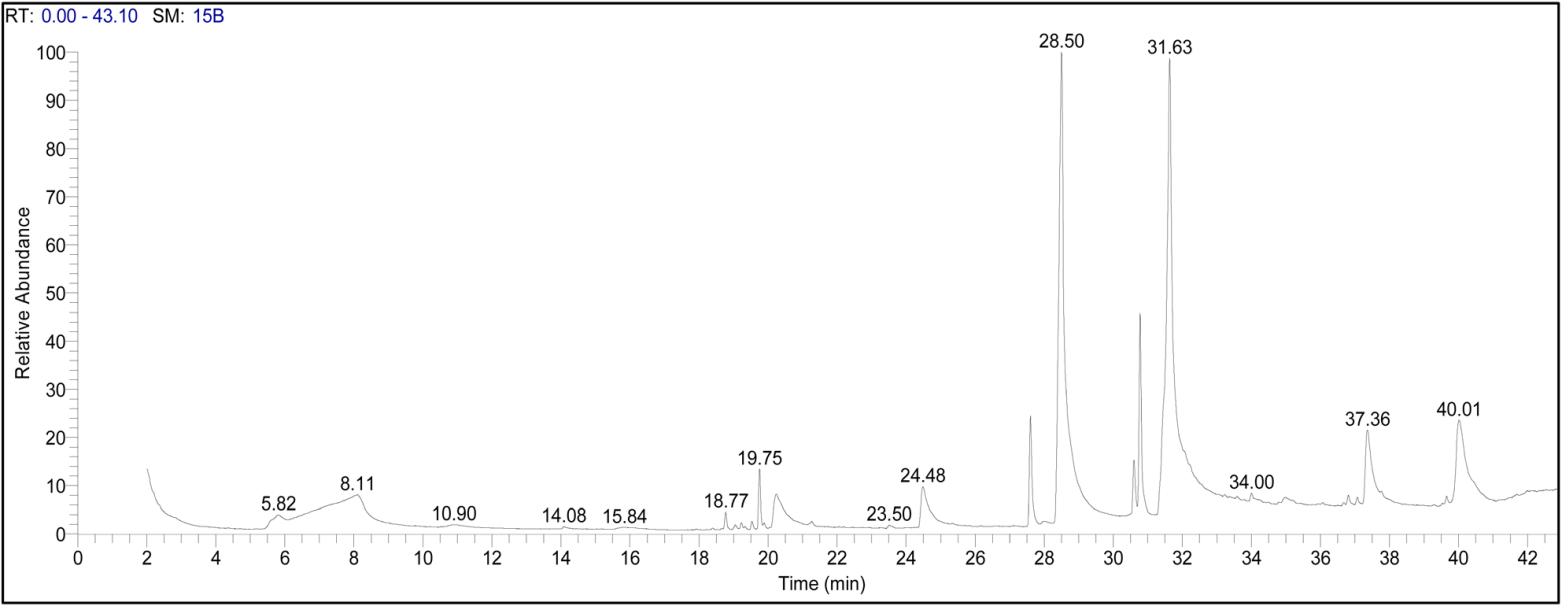
The GC-MS chromatogram of crude methanolic ASH extract.

**Fig. 3:**
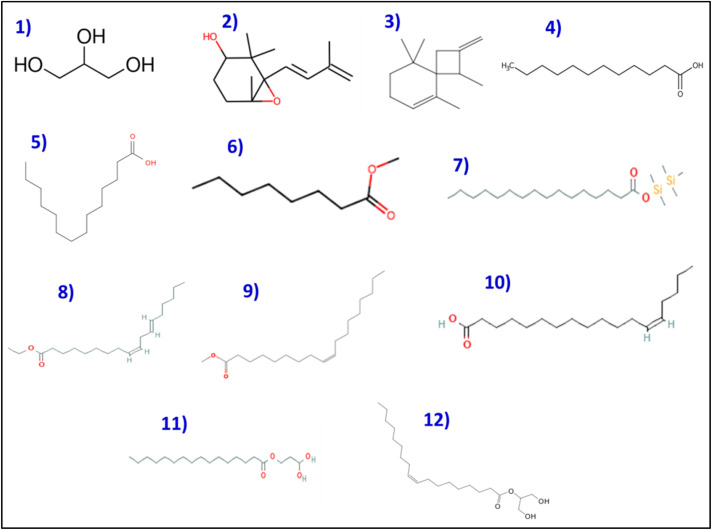
The bioactive molecules identified using GC-MS analysis of crude methanolic ASH extract. The labeled chemical structures (1–12) correspond to the compounds listed in Table 2.

The GC-MS analysis of the ASH extract has been found to possess a diversified composition of 12 bioactive molecules (Table 2). The GC-MS profiling of the 12 peaks of the identified bioactive molecules was illustrated in Fig. 2 and 3. The whole chemical profile shows the presence of fatty acids, glycerol-based lipids, terpenoids, and steroid-like moieties (Fig. 3). The presence of appreciable amounts of fatty acids, namely *n*-hexadecanoic acid (30.42%) and *cis*-13-octadecenoic acid (31.68%), shows that the ASH extract is a good source of lipids. These fatty acids and their esters, *viz.* hexadecanoic acid methyl ester and ethyl(9*Z*,12*Z*)-9,12-octadecadienoate are well-reported for their biological activities, including anti-inflammatory, antioxidant, and antimicrobial properties. The existence of 1,2,3-propanetriol (glycerol) and hexadecanoic acid, 1-(hydroxymethyl)-1,2-ethanediyl ester (a glyceride) suggests that the extract is abundant in glycerol-based lipids. The identification of compounds such as 2,2,6-trimethyl-1-(3-methylbuta-1,3-dienyl)-7-oxabicyclo[4.1.0]heptan-3-ol and 1,5,9,9-tetramethyl-2-methylene-spiro[3.5]non-5-ene points to the presence of terpenoid or steroid-like structures in the extract. Terpenoids and steroids are known for their diverse biological activities, including anti-inflammatory, adaptogenic, and neuroprotective effects.

**Table 2:** GC-MS profiling of crude methanolic ASH extract^a^

No.	RT	Compound names	MW	MF	Area%
1	8.12	1,2,3-Propanetriol	929	C_3_H_8_O_3_	2.31
2	18.77	2,2,6-Trimethyl-1-(3-methylbuta-1,3-dienyl)-7-oxabicyclo[4.1.0]heptan-3-ol	787	C_14_H_22_O_2_	0.51
3	19.75	1,5,9,9-Tetramethyl-2-methylene-spiro[3.5]non-5-ene	812	C_14_H_22_	1.57
4	20.23	Dodecanoic acid	931	C_12_H_24_O_2_	3.45
5	24.48	Tetradecanoic acid	927	C_14_H_28_O_2_	3.47
6	27.59	Hexadecanoic acid, methyl ester	923	C_17_H_34_O_2_	3.80
7	28.51	*n*-Hexadecanoic acid	940	C_16_H_32_O_2_	30.42
8	30.60	Ethyl(9*z*,12*z*)-9,12-octadecadienoate	911	C_20_H_36_O_2_	1.78
9	30.77	9-Octadecenoic acid, methyl ester, (*E*)-	924	C_19_H_36_O_2_	6.34
10	31.64	*cis*-13-Octadecenoic acid	922	C_18_H_34_O_2_	31.68
11	37.36	Hexadecanoic acid, 1-(hydroxymethyl)-1,2-ethanediyl ester	799	C_35_H_68_O_5_	5.85
12	40.01	9-Octadecenoic acid (*Z*)-, 2-hydroxy-1-(hydroxymethyl)ethyl ester	826	C_21_H_40_O_4_	8.82

a RT = retention time (min); MW = molecular weight; MF = molecular formula.

Likewise, *n*-hexadecanoic acid, a saturated fatty acid, is known for its strong antimicrobial properties due to its ability to integrate into microbial lipid bilayers, disrupting membrane integrity and leading to increased permeability. This disruption compromises essential cellular functions such as ion homeostasis, nutrient transport, and enzyme activity, ultimately causing microbial cell lysis and death. Furthermore, *n*-hexadecanoic acid interferes with microbial quorum sensing pathways, limiting biofilm formation and inhibiting bacterial communication, which is crucial for pathogenicity and resistance mechanisms.^36,37^

In addition to *n*-hexadecanoic acid, other bioactive components, including *cis*-13-octadecenoic acid and various steroidal lactones (withanolides), contribute to antimicrobial activity by targeting membrane fluidity, inhibiting essential enzymes, and generating oxidative stress within microbial cells.^38^ The synergistic effect of these compounds enhances the overall antimicrobial potency of ASH extract, making it an effective additive in Cs-based films for food preservation.

The antimicrobial properties of extract sourced from ASH have been acknowledged for their significant value in combating foodborne pathogens and preserving food. In the present study, the methanolic extract of ASH contains various bioactive compounds, including alkaloids, steroidal lactones (withanolides), saponins, and flavonoids, which contribute to its effectiveness against microbes. Phytochemical studies of extract sourced from ASH have revealed diverse chemical components, including steroidal compounds, alkaloids, phenolic compounds, saponins with additional acyl groups, and withanolides with glucose at carbon 27.^39,40^ Over 12 alkaloids, approximately 40 withanolides, and multiple sitoindosides have been documented in ASH's aerial parts, roots, and berries. The roots contain alkaloids, amino acids, steroids, volatile oils, starch, reducing sugars, glycosides, hentriacontane, dulcitol, and withaniol. Phytochemical research has shown that these chemical constituents hold significant potential for further study and therapeutic applications.^41^ This wide array of compounds demonstrates the rich complexity within ASH, making it a fascinating subject for continued exploration and analysis within phytochemistry.^42^ Further, high concentrations of fatty acids, such as *n*-hexadecanoic acid and *cis*-13-octadecenoic acid, disrupt microbial cell membranes, leading to cell lysis and death. Additionally, terpenoids and steroidal lactones, like withaferin A, inhibit microbial protein and enzyme synthesis, which are essential for pathogen survival.^43^

### Antioxidant properties of ASH extract

**Fig. 4:**
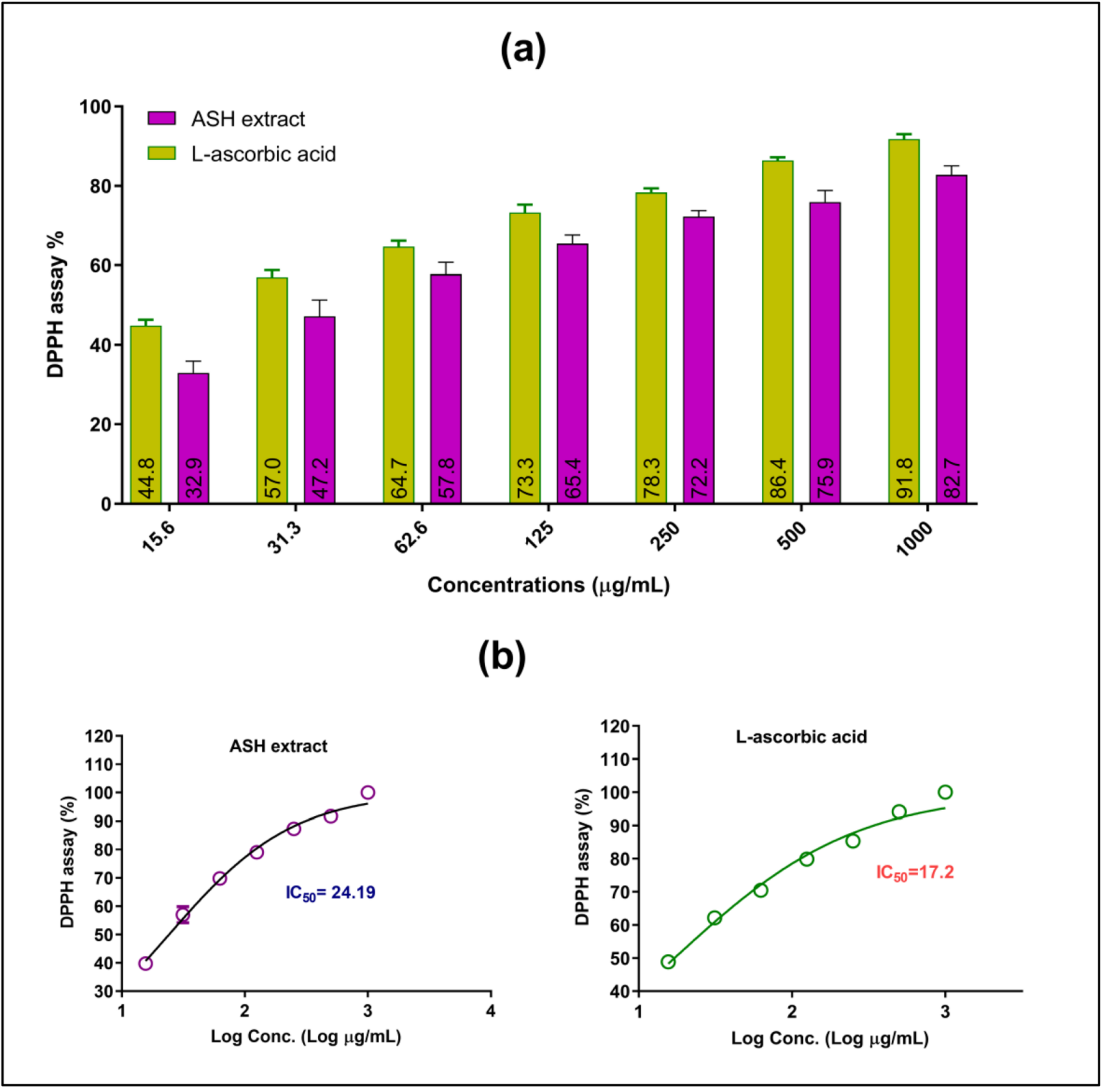
(a) Effect of dose varying-response of ASH extract and l-ascorbic acid on the % inhibition of DPPH radical scavenging, (b) IC_50_ for scavenging DPPH free radicals of ASH extract and l-ascorbic acid. Values represented as mean ± SD.

The antioxidant potential of ASH extract was explored using of DPPH radical scavenging method. Fig. 4(a and b) presents the results of the DPPH radical scavenging activity of ASH extract and l-ascorbic acid (vitamin C) at various concentrations from 15.5–1000 μg mL^−1^. Fig. 4(a) displays the percentage of DPPH inhibition against the concentration (μg mL^−1^) of both ASH extract and l-ascorbic acid. It shows that both substances exhibit increasing DPPH radical scavenging activity with rising concentrations. Notably, the ASH extract demonstrates a higher %DPPH inhibition of 82.7%. However, at higher concentrations (100 μg mL^−1^ and above), 91.8% of % DPPH inhibition was recorded.

In addition, Fig. 4(b) illustrates the dose–response curves for the ASH extract and l-ascorbic acid, enabling the calculation of their IC_50_ values, which indicate the concentration needed to inhibit 50% of the DPPH radicals. The IC_50_ value for the ASH extract is 24.19 μg mL^−1^, reflecting its potent antioxidant activity. In contrast, the IC_50_ value for l-ascorbic acid is 17.2 μg mL^−1^, suggesting a slightly higher antioxidant capacity than the ASH extract. It is important to note that, both the ASH extract and l-ascorbic acid possess strong DPPH radical scavenging abilities. The ASH extract shows higher antioxidant activity at lower concentrations, while l-ascorbic acid becomes more potent at higher concentrations. The IC_50_ values provide a quantitative measure of their comparative antioxidant potencies.

Our results are in accordance with Ganguly *et al.*,^44^ who stated that the hydromethanolic extracts of ASH roots displayed noteworthy antioxidant properties, with an IC_50_ value of more than 30 μg mL^−1^ in DPPH radical scavenging activity. In a study by Dhanani *et al.*,^45^ it was found that the antioxidant activity of aqueous extracts from ASH roots ranged from 0.40 to 1.20 mg mL^−1^ (IC_50_) in terms of DPPH values. Meanwhile, Chaudhary *et al.*^46^ reported a higher DPPH value of 4612.17 μg mL^−1^ (IC50), and Ganguly *et al.*^44^ obtained even lower values of DPPH at 111.31 μg mL^−1^ (IC_50_). These results emphasize the significant variability in the antioxidant activity of ASH extract, as evidenced by the DPPH values.

Antioxidant mechanisms are primarily classified into two fundamental pathways: electron transfer (ET) and hydrogen atom transfer (HAT). The ET mechanism involves the donation of an electron from an antioxidant molecule to neutralize free radicals, stabilizing them and preventing oxidative damage. This process is particularly significant in polyphenols and flavonoids found in ASH extract, as their conjugated aromatic structures allow for the effective delocalization of unpaired electrons, thereby reducing oxidative stress.^47,48^ The ET mechanism is prominently observed in the DPPH radical scavenging assay, where antioxidants from ASH extract reduce DPPH radicals by donating electrons, leading to a visible color change that quantifies antioxidant capacity.^49^

Conversely, the HAT mechanism functions by transferring a hydrogen atom from an antioxidant molecule to a reactive radical species, effectively neutralizing it. Fatty acids present in ASH extract, such as *n*-hexadecanoic acid and *cis*-13-octadecenoic acid, play a critical role in this process by donating hydrogen atoms to lipid peroxyl radicals, which terminates lipid peroxidation chain reactions.^50^ This activity is essential in preventing oxidative degradation of lipids in food matrices, thereby enhancing the shelf life of perishable products.

### Exploring antimicrobial activity

**Fig. 5:**
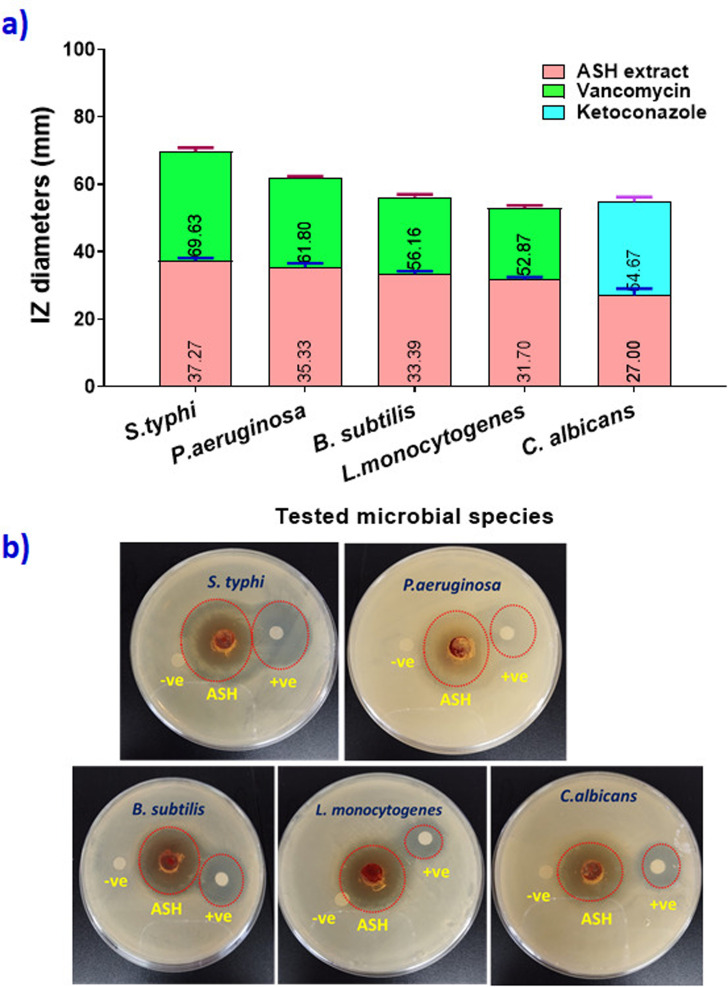
(a) Diameters of inhibition zones (in mm) of ASH extract against different microbial species, (b) photographic images of agar plates that used in agar well diffusion assay.

The antimicrobial effect of ASH extract against five microbial strains including, *S. typhie*, *P. aeruginosa*, *B. subtilis*, *L. monocytogenes* and *C. albicans* was estimated using agar well diffusion assay. Fig. 5 presents the diameters of inhibition zones (in mm) for different bacterial species when exposed to ASH extract, Vancomycin (for bacterial strains), and Ketoconazole (for yeast), offering a comparative assessment of their antimicrobial effects. Notable findings from the graph indicate that the ASH extract displays the largest inhibition zone (37.27 mm) against *S. typhi*, indicating a potent antimicrobial impact on this species. Moreover, the inhibition zones of ASH against *P. aeruginosa*, *B. subtilis*, *L. monocytogenes* and *C. albicans* were 35.33 mm, 33.39 mm, 31.70 mm, and 27 mm, respectively. Vancomycin, a conventional antibiotic, demonstrates the most substantial inhibition zones against *P. aeruginosa*, *B. subtilis*, and *L. monocytogenes*, indicating its superior efficacy against these bacterial strains. Ketoconazole, an antifungal medication, exhibits the largest inhibition zone against *C. albicans*, showcasing its potent antifungal properties. The variations in inhibition zones among different bacterial species underscore the specific antimicrobial characteristics of the ASH extract, Vancomycin, and Ketoconazole. This emphasizes the significance of evaluating a variety of pathogens to determine the broad-spectrum antibacterial efficacy of these compounds. The antibacterial effects of ASH extract originate from many bioactive components, including with anolides, alkaloids, and phenolic compounds. Furthermore, these compounds can disrupt the cell membranes of microbes, resulting in cell lysis and death. Withanolides also have the ability to inhibit microbial protein synthesis and enzyme activities, which further hinders the growth and survival of microbes. The combined effects of these bioactive compounds make ASH extract a powerful antimicrobial agent that can be applied in multiple fields, including medicine, agriculture, and food preservation. Murugan *et al.*,^51^ reported identified ASH root extract as the primary active component in ASH extract that demonstrated antimicrobial activity against various bacteria. However, Ha *et al.*^52^ reported that withanolide glycosides are the main bioactive compounds found in the roots of ASH. Mehta *et al.*,^53^ additionally emphasized significant differences in the antimicrobial efficacy of different ASH extracts, indicating that the specific bioactive compounds responsible for antimicrobial activity in ASH may depend on the part of the plant used and the extraction method employed.

The antimicrobial activity of ASH extract against *S. typhi*, *P. aeruginosa*, *B. subtilis*, *L. monocytogenes*, and *C. albicans* is primarily attributed to its bioactive compounds, including *n*-hexadecanoic acid, *cis*-13-octadecenoic acid, and withanolides. These compounds exert their effects through multiple mechanisms, including membrane disruption, inhibition of protein synthesis, and oxidative stress induction. For *S. typhi*, ASH extract disrupts membrane integrity, leading to increased permeability and leakage of intracellular contents.^19^ In *P. aeruginosa*, the extract interferes with quorum sensing, preventing bacterial communication and biofilm formation. For *B. subtilis*, ASH-derived compounds inhibit peptidoglycan synthesis, compromising cell wall integrity. In *L. monocytogenes*, the antimicrobial activity is linked to ATP synthesis inhibition and ion imbalance, leading to cell death. Against *C. albicans*, ASH extract disrupts ergosterol biosynthesis, impairing fungal membrane stability and inducing oxidative stress.^22,54,55^ These mechanisms collectively highlight the broad-spectrum antimicrobial potential of ASH extract, supporting its application in food preservation and active packaging.

### Determination of MIC and MBC values

**Fig. 6:**
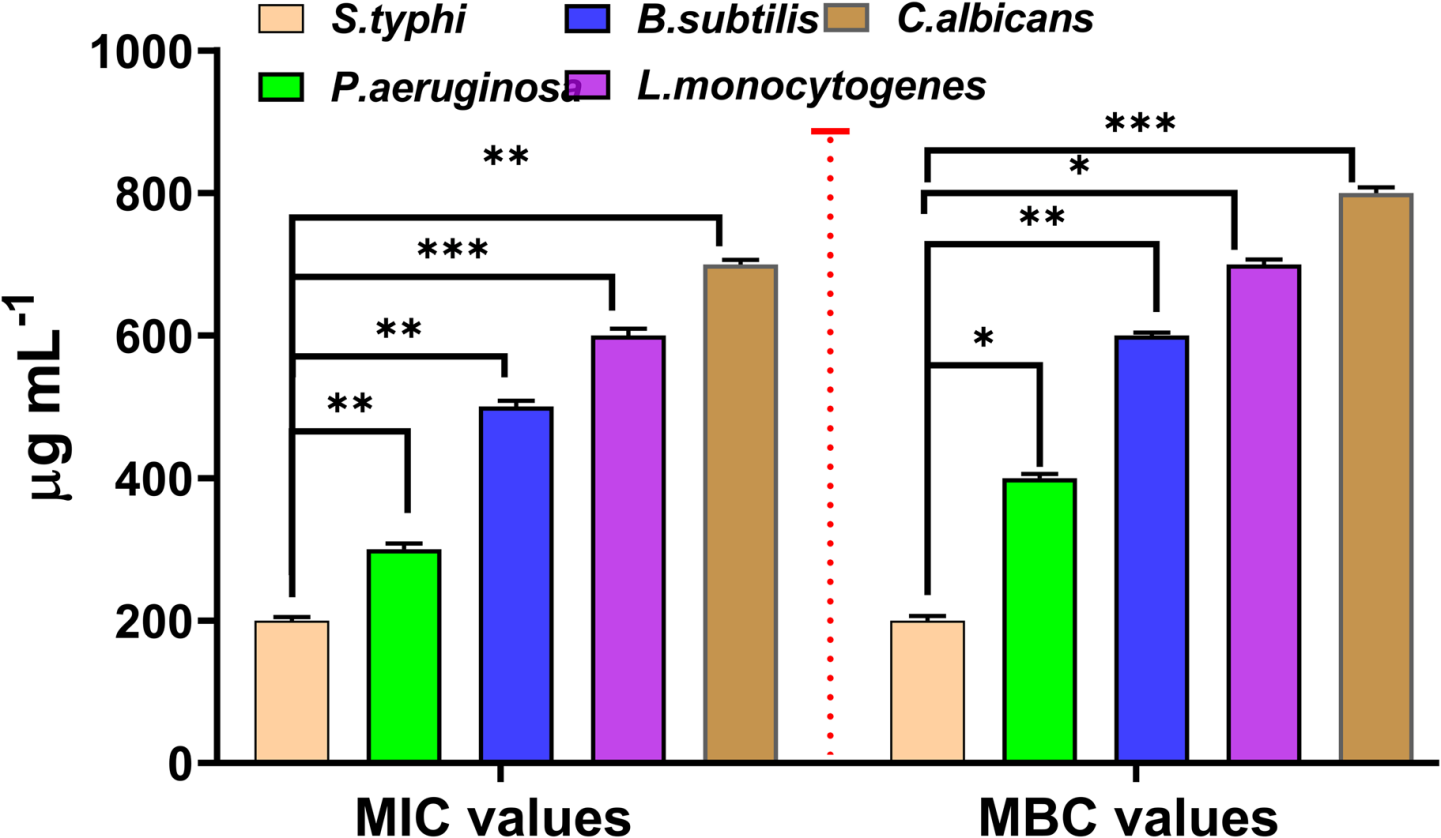
Expected MIC and MBC values (μg mL^−1^) of ASH extract against tested microbial strains. Data are presented as mean ± SD. Statistical significance was determined using a one-way ANOVA test. * indicates *P* value < 0.001, ** indicates *P* value < 0.0001, and *** indicates *P* value < 0.00001.

The effectiveness of the ASH extract as an antimicrobial was assessed by determining the MIC and MBC against a range of microorganisms using the resazurin dye method (Fig. 6). The MIC for *S. typhi* was found to be 200 μg mL^−1^, indicating the concentration needed to inhibit its growth, with the MBC also being 200 μg mL^−1^, showing the concentration required to kill this pathogen. These results demonstrate the high susceptibility of *S. typhi* to the ASH extract. As for *P. aeruginosa*, the MIC was 300 μg mL^−1^ and the MBC was 400 μg mL^−1^, indicating that a slightly higher concentration is needed for bactericidal effects compared to inhibitory effects. The lowest concentration at which *B. subtilis* is inhibited (MIC) was determined to be 500 μg mL^−1^, with a MBC of 600 μg mL^−1^. Likewise, *L. monocytogenes* showed an MIC of 600 μg mL^−1^ and an MBC of 700 μg mL^−1^, while *C. albicans* displayed an MIC of 700 μg mL^−1^ and an MBC of 800 μg mL^−1^.

These results indicate the necessity of higher concentrations to effectively prevent and eliminate these microorganisms, particularly the fungal strain. Assessing the MIC and MBC values is vital in evaluating the antimicrobial efficacy of natural ASH extracts. The MIC is the lowest concentration needed to inhibit visible microbial growth, whereas the MBC is the lowest concentration required to eliminate 99.9% of the population. These values demonstrate the extract's diverse effectiveness against different pathogens. These results underscore the extensive antibacterial efficacy of ASH extract, indicating its potential as a natural antimicrobial agent. The differences in MIC and MBC values across diverse bacteria highlight the need of assessing both metrics to comprehensively grasp efficacy and dose requirements for varied diseases. This investigation supports the use of ASH extract in combating microbiological infections and improving food safety.

The results of the present study indicated that the bioactive compounds in ASH extract possess significant antimicrobial and antioxidant activities. Incorporating these extracts of Cs can lead to the development of bioactive packaging materials that enhance food preservation and reduce microbial contamination. Similarly, date palm residues, widely available as agricultural waste after fruit harvesting in Middle Eastern regions, offer a sustainable and eco-friendly source for developing packaging films and coatings, reducing the need for petroleum-based plastics. Utilizing agro-waste from date palm processing, particularly date seeds offers a sustainable approach to extracting natural compounds with bio-functional properties. These compounds can be blended with biodegradable or synthetic polymers to produce active packaging films with improved shelf-life and microbial resistance.^56,57^

Moreover, the studies have demonstrated that withanolides exhibit bactericidal effects by disrupting bacterial membrane integrity, increasing membrane permeability, and causing leakage of intracellular contents, ultimately leading to cell lysis. *n*-Hexadecanoic acid and *cis*-13-octadecenoic acid, two primary fatty acids found in ASH extract, integrate into microbial lipid bilayers, destabilizing the membrane structure and affecting ion transport. The resulting loss of membrane integrity contributes to bacterial cell death. As a selectively permeable barrier, the bacterial cell membrane controls the flow of materials between the extracellular and intracellular spaces to preserve conditions necessary for cellular functions. Maintaining membrane integrity is essential for bacterial survival, since even little disturbances may hinder metabolic activities, including enzymatic activity. Consequently, evaluating membrane integrity yields significant insights into the antibacterial mode of action. Studies have demonstrated that monitoring specific cell leakage markers, such as the absorbance of nucleic acids at 260 nm and the protein content released into the bacterial supernatant, is a reliable indicator of membrane disruption. A significant increase in these markers compared to untreated cells suggests membrane damage caused by exposure to an antimicrobial agent.^58,59^

### Characterizations of films

**Fig. 7:**
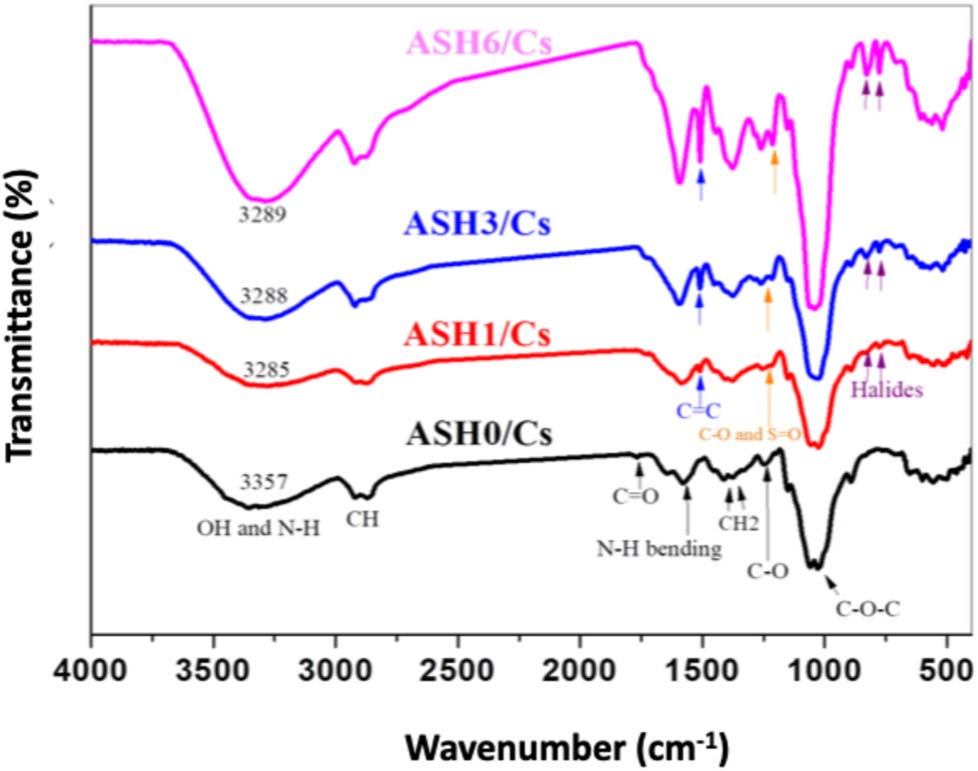
FTIR of ASH0/Cs, ASH1/Cs, ASH3/Cs and ASH6/Cs films.

The FTIR spectra of pure Cs film (ASH0/Cs) and Cs loaded with different concentrations of ASH extract (ASH1/Cs, ASH3/Cs, and ASH6/Cs) are illustrated in Fig. 7. For ASH0/Cs, there are specific peaks were assigned. The peak at 3357 cm^−1^ is due to the stretching vibration of combined hydroxyl and amino groups. The appeared peaks at 2918 and 2871 cm^−1^ are ascribed to the asymmetric and symmetric stretching modes of saturated C–H groups. The weak peak at 1767 cm^−1^ is assigned to C=O of acetate groups. The peak at 1578 cm^−1^ is ascribed to N–H bending. Meanwhile, the peaks at 1411 and 1377 cm^−1^ are attributable to CH_2_ deformation, and the stretching mode of the glucosidic ring extended between 1248–800 cm^−1^.^60^ ASH extract is a polyphenolic compound that contains many active groups such as alcoholic and phenolic OH, C=C, C=O, C–H, *etc.* Different concentrations of ASH extract were added to Cs. Regarding the FTIR of ASH1/Cs (Cs contains a lower concentration of ASH, 1 mL), OH become less intensity, broader, and shifted to 3285 cm^−1^, affirming the formation of more hydrogen bonds. For ASH3/Cs film (3 mL of ASH extract) and ASH6/Cs film (6 mL of ASH extract), the intense of OH enhanced as the extract is a polyphenolic compound contains many alcoholic and phenolic OH that overlapped with OH of Cs. A New peak appeared at 1515 cm^−1^ is due to the C=C stretching, and at 777 and 702 cm^−1^ which may be attributed to the halide groups of ASH extract.^61,62^

**Fig. 8:**
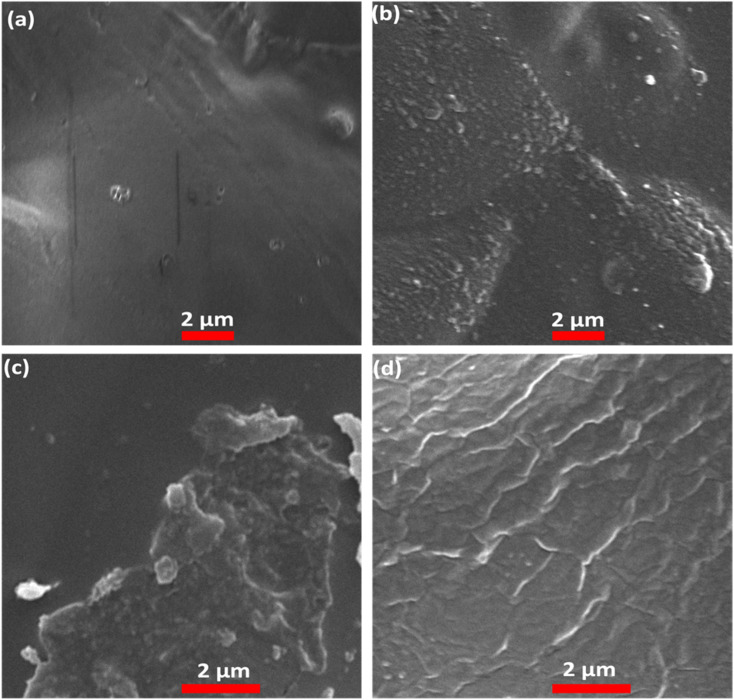
SEM of (a) ASH0/Cs, (b) ASH1/Cs, (c) ASH3/Cs, and (d) ASH6/Cs.

The surface structure of ASH0/Cs, ASH1/Cs, ASH3/Cs, and ASH6/Cs films is displayed in Fig. 8. It can be seen that pure Cs film has a smooth surface (ASH0/Cs, Fig. 8(a)), which turned into a rough surface when the film was composed of Cs and ASH extract. Fig. 8(b–d) illustrates the rough surface of ASH1/Cs, ASH3/Cs, and ASH6/Cs films. The change in surface appearance from smooth to rough is ascribed to the addition of ASH to the Cs solution. The deposition of ASH molecules onto the outer surface of the film can be attributed to the relatively poor miscibility between Cs and the ASH extract.

**Fig. 9:**
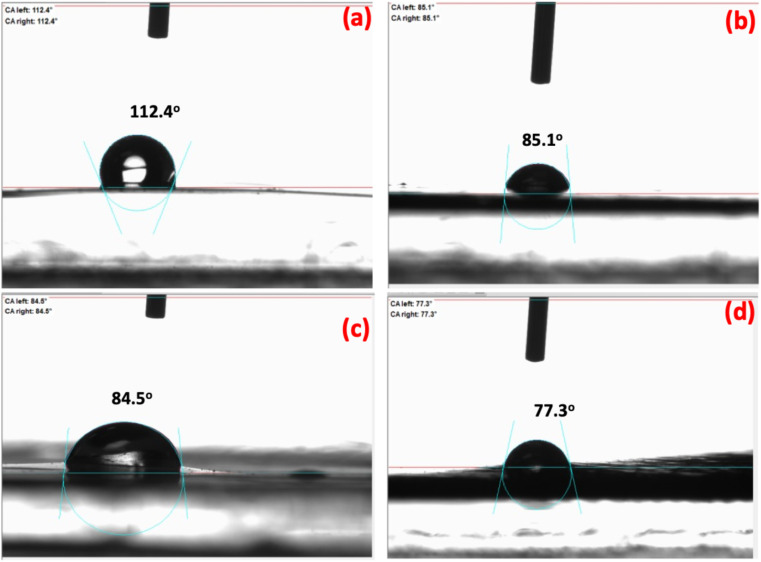
WCA of (a) ASH0/Cs, (b) ASH1/Cs, (c) ASH3/Cs, and (d) ASH6/Cs.

Fig. 9 presented the WCA of the prepared Cs films with and without ASH loading; ASH0/Cs, ASH1/Cs, ASH3/Cs and ASH6/Cs. As mentioned above, ASH0/Cs is a code for pure Cs film and without ASH extract. The other codes are presented for the different ratios of Cs and ASH that used for film formation. From Fig. 9, it was observed that, the WCA values of ASH0/Cs, ASH1/Cs, ASH3/Cs and ASH6/Cs were 112.4°, 85.1°, 84.5°, and 77.3°, respectively. As shown, the high WCA of pure Cs film (ASH0/Cs). The addition of ASH extract leads to decreasing the WCA values of Cs films. As the concentration of ASH increased with decreasing the concentration of Cs, the WCA was decreased. The WCA value was significantly reduced to 77.3° when the concentration of Cs/ACH was 6 mL/24 mL of Cs. The decreasing value signifying the hydrophilicity of the formed films. ASH extract contains alcoholic and phenolic compounds which able to increase the hydrophilicity of the films.

Overall, the addition of ASH enhanced WCA of the produced films and enhanced the hydrophilicity properties of the films. All WCA values for the films loaded with ASH are less than 90° which affirming the hydrophilicity of Cs films.

Water vapor transmission rate (WVTR) and oxygen permeability (OP) parameters are very important for determining the quality of the food packaging films. The lower the WVTR value, the more suitable the film is for preserving and packaging preserved foods. It was found that Cs film (ASH0/Cs) has a relatively high value of WVTR (3.35 ± 0.11 × 10^−5^ g mm^−2^ h^−1^) when compared to the Cs film that contains different concentrations of ASH extract. The WVTR values of ASH1/Cs, ASH3/Cs and ASH6/Cs were found to be 3.33 ± 0.09 × 10^−5^, 3.31 ± 0.03 × 10^−5^ and 2.94 ± 0.07 × 10^−5^ g mm^−2^ h^−1^.

The oxygen permeability (OP) of ASH0/Cs, ASH1/Cs, ASH3/Cs and ASH6/Cs films. It was observed that the OP of Cs film is 10.24 ± 0.51 (cm^3^ μm per m^2^ per day per kPa) and marginally decreased with increasing the concentration of ASH to 1 mL (10.07 ± 0.73 cm^3^ μm per m^2^ per day per kPa). The OP of ASH3/Cs and ASH6/Cs was significantly decreased with increasing the concentration of ASH extract to 3 and 6 mL and became 9.75 ± 0.46 (cm^3^ μm per m^2^ per day per kPa) and 7.26 ± 0.34 (cm^3^ μm per m^2^ per day per kPa).

### Biological assessment of Cs films

The present work included the integration of ASH extracts with Cs film to create effective food packaging materials and prolong the shelf life of perishable commodities by inhibiting the proliferation of foodborne microbes. Three distinct ratios of ASH extract were introduced into Cs film, creating films labeled ASH0/Cs (control without ASH extract), ASH1/Cs, ASH3/Cs, and ASH6/Cs. The different concentrations were employed to evaluate the effectiveness of ASH extract in improving the antibacterial characteristics of the Cs films, enabling a novel approach to food preservation.

### Phytochemical contents and total antioxidant capacity

Three Cs films were examined after loading ASH extract after loading with different ratios of ASH extract (ASH1/Cs, ASH3/Cs, and ASH6/Cs) to determine the extent of sustained and effective release of photochemical content. Total phenolic content (TPC), total flavonoid content (TFC), and total antioxidant capacity (TAC) in Cs films loaded with ASH extract were quantified using the data shown in Table 3. Higher ASH extract loading significantly increased the TPC values. The TPC values for ASH1/Cs, ASH3/Cs, and ASH6/Cs were 27.51 ± 0.34, 45.18 ± 0.96, and 68.36 ± 0.48 mg GAE per 100 g, respectively. The continuing increase in phenolic content in the Cs films may be attributed to increased ASH extract concentrations. Due to the significant antioxidant qualities of phenolic compounds, it is possible that films with greater ASH extract ratios have more potential for antioxidant activity. Likewise, increased ASH extract loading was associated with higher TFC values. TFC values for ASH1/Cs, ASH2/Cs, and ASH3/Cs were 16.36 ± 0.72 mg QE per 100 g, 21.27 ± 0.28 mg QE per 100 g, and 34.05 ± 0.61 mg QE per 100 g, respectively. The extract's antioxidant action was attributed chiefly to flavonoids, which also provided oxidative stress protection. Superior antioxidant qualities were shown by the greater flavonoid concentration in films with higher ASH extract ratios. The TAC values, which indicate the improved antioxidant capacity with greater ASH extract loading, were further confirmed by the expression of the results in mg of ascorbic acid equivalents per 100 g of extract (mg AAE per 100 g extract).

**Table 3:** Quantification of TPC, TFC, and TAC of Cs films after loading with various ratios of ASH extract

Parameters	Unit	Tested Cs films after loading with ASH extract		
		ASH1/Cs	ASH3/Cs	ASH6/Cs
TPC	mg GAE per 100 g extract	27.51 ± 0.34	45.18 ± 0.96	68.36 ± 0.48
TFC	mg QE per 100 g extract	16.36 ± 0.72	21.27 ± 1.28	34.05 ± 0.61
TAC	mg AAE per 100 g extract	43.65 ± 1.02	59.82 ± 1.53	73.15 ± 2.28

TAC values for ASH1/Cs were 43.65 ± 1.02 mg AAE per 100 g, 59.82 ± 1.53 mg AAE per 100 g for ASH3/Cs, and 73.15 ± 2.28 mg AAE per 100 g for ASH6/Cs. These values showed that when ASH extract concentrations increase, the films' capacity to scavenge free radicals increases as well, strengthening their protective qualities. The TPC, TFC, and TAC values progressively rose with the increasing ratio of ASH extract in the Cs films, indicating that a higher concentration of ASH extract enhances the films' antioxidant properties. The enhancement is attributed to elevated levels of phenolic and flavonoid compounds, renowned for their antioxidant capabilities. The findings indicate that Cs films infused with ASH extract may be very effective in applications requiring antioxidant and antibacterial properties, such as food packaging, where they enhance shelf life and quality.

### Antimicrobial potential of films

Cs's film-forming capability allows it to create thin layers with specific thickness and strength under controlled conditions. Extracted from crustacean shells like shrimp and crab.^63^ Cs is a natural polysaccharide known for its strong film-forming properties, which stem from its unique molecular structure and chemical characteristics. Its abundant hydroxyl and amino groups form hydrogen bonds with water molecules, allowing Cs to become a colloidal solution in aqueous environments. As water evaporates, the hydrogen bonds between Cs molecules strengthen, leading to a film with defined strength and elasticity.^64^

The antimicrobial efficacy of Cs films incorporating different concentrations of ASH extract was evaluated against a diverse range of microorganisms, such as *S. typhi*, *P. aeruginosa*, *B. subtilis*, *L. monocytogenes*, and *C. albicans*. The results indicated that the addition of ASH extract significantly enhanced the antibacterial efficacy of the Cs films. The inhibitory zones for *S. typhi* exhibited a substantial enlargement, progressing from 0 mm in the control group lacking ASH to 34 mm, 35 mm, and 35.49 mm, respectively, in the groups treated with ASH1/Cs, ASH3/Cs, and ASH6/Cs. The values for these parameters were extracted from Cs films infused with ASH extract in escalating concentrations. The findings indicate that incorporating ASH extract into Cs films significantly enhances their antibacterial properties, with the most effective concentration (ASH6/Cs) demonstrating the greatest efficacy. The enhanced antibacterial activity of ASH extract can be ascribed to bioactive constituents such as flavonoids, phenolic compounds, and withanolides. These compounds rupture the cell walls and membranes of microbes, leading to cellular demise. This research provides evidence that ASH-loaded Cs films may be utilized to improve the safety and preservation of food during packaging. These modified Cs films effectively extend the storage life of perishable products by inhibiting the growth of microorganisms.

**Fig. 10:**
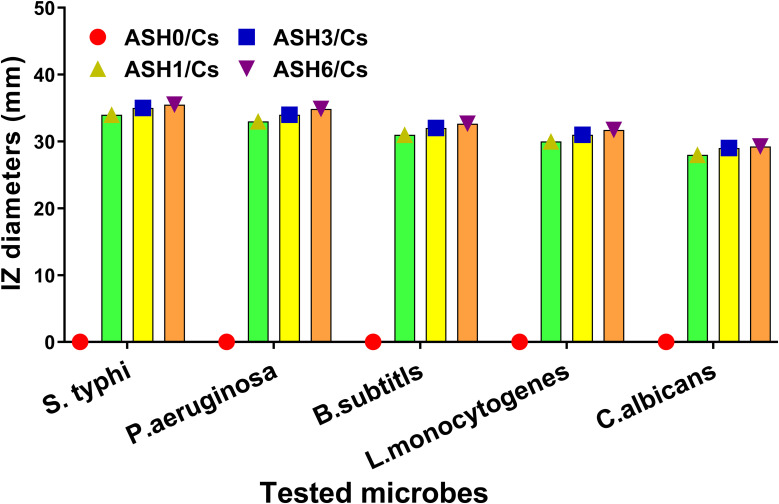
The antimicrobial effect and the inhibition zone diameters (mean ± SD) of tested Cs films before and after loading with ASH extract against some foodborne pathogens.

Fig. 10 illustrates the antimicrobial properties of Cs films loaded with ASH extract at different concentrations against a range of foodborne pathogens. The significant results indicated that Cs films without ASH extract loading (ASH0/Cs) demonstrated no antimicrobial activity against all microorganisms tested, as evidenced by the absence of 0 mm inhibition zones. Conversely, when ASH extract was incorporated into Cs films, a significant antimicrobial effect was observed against every foodborne pathogen examined. In particular, the inhibition zone diameters of Cs films loaded with ASH extract (ASH1/Cs, ASH3/Cs, and ASH6/Cs) increased in direct proportion to the concentration of ASH extract. As an illustration, the inhibition zone diameters observed for *S. typhi* varied between 34 mm (ASH1/Cs) and 35.49 mm (ASH6/Cs), suggesting a substantial degree of antimicrobial efficacy against this particular pathogen. Comparable patterns were noted concerning additional bacterial pathogens, including *B. subtilis* (30 mm to 31.64 mm), *P. aeruginosa* (33 mm to 34.85 mm), and *L. monocytogenes* (30 mm to 31.71 mm). Furthermore, as ASH extract concentrations increased, the obstruction zones of the fungal pathogen *C. albicans* increased from 28 mm to 29.25 mm. The findings of this study indicate that the antimicrobial properties of Cs films are substantially improved when loaded with ASH extract. As a result, these films could potentially be utilized in food packaging or other sectors that necessitate antimicrobial protection. The observed rise in inhibition zone diameters in a dose-dependent manner suggests that manipulating the concentration of ASH extract can enhance the films' antimicrobial effectiveness. The results reveal significant insights regarding the broad-spectrum antimicrobial effectiveness of Cs films loaded with ASH extract against various foodborne pathogens. This underscores their capacity to function as an organic and efficacious antimicrobial system, thereby augmenting food safety and prolonging the expiration life of perishable goods.

### Toxicity assessment of explored films

**Fig. 11:**
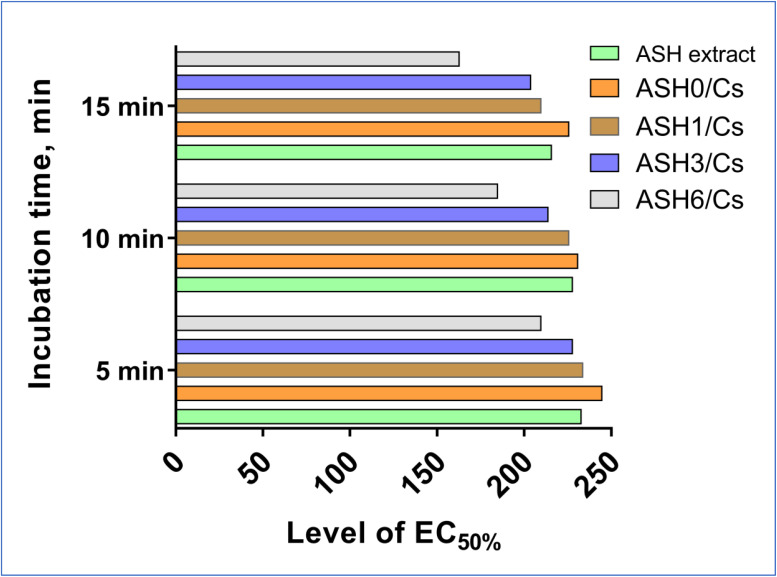
The measured toxicity level and the assessed EC_50%_ values of ASH extract and films (ASH0/Cs, ASH1/Cs, ASH3/Cs, and ASH6/Cs).

The EC_50%_ values, which represent the effective concentration causing 50% toxicity, and toxicity levels of ASH extract and its Cs films at various incubation periods are depicted in Fig. 11. These figures offer significant insights into the safety profiles of the films. All tested films had EC_50%_ values greater than 100, indicating that they are biocompatible, non-toxic, and safe for use as food packaging materials, according to the results. In toxicological studies, the EC_50%_ value is a crucial metric that indicates the concentration of a substance that induces 50% toxicity in the organisms under investigation. An EC_50%_ value exceeding 100 designates a substance as non-toxic, thereby serving as a substantial safety criterion for food-contact materials. Both the ASH extract and the Cs films laden with ASH extract are non-toxic, as indicated by the high EC_50%_ values. This finding suggests that the utilisation of these materials in food packaging does not present any substantial health hazards, an essential factor in guaranteeing the safety of consumers. By virtue of their safety profile, the ASH extract-loaded Cs films exhibit biocompatibility. This implies that the films have the capability to interact non-hazardously with food products, thereby rendering them appropriate for use in packaging applications. The findings validate the safety of employing Cs films laden with ASH extract for food packaging. By preventing the discharge of potentially hazardous substances that may contaminate food, the materials ensure that the packaged products retain their quality and safety.

Plant polyphenols, such as tea polyphenols, can enhance the physicochemical properties of films by forming cross-linkages. The polyphenols include phenolic acids, anthocyanins, catechins, and flavonoids, and they are commonly used in food preservation.^65^ A study has explored the incorporation of polyphenols into Cs-based edible films.^66^ However, their impact on Cs-based packaging has not been comprehensively reviewed, particularly in terms of improvements in physicochemical properties, bioactivity for food preservation, and nutritional value.

### Preservative performance of ASH3/Cs film on fresh-cut strawberries

Fruits are nutrient-rich, but their exposure to the external environment promotes bacterial growth, making them susceptible to spoilage during storage and transportation, ultimately affecting quality or leading to decay.^67^ Cs is widely used in fruit preservation through two main approaches: film wrapping and coating.^68^ In coating, Cs is applied *via* spraying, brushing, or dipping, forming a thin protective layer on the fruit surface to maintain freshness. In film wrapping, Cs's film-forming ability creates a wrap around the fruit, acting as a barrier that enhances preservation.

The efficiency of the ASH3/Cs film in keeping the fresh-cut strawberries free from deterioration was assessed. The film was mainly selected for its character development, which is of high quality, and its anti-bacterial properties. Strawberry fruits were wrapped in PE plastic, ASH3/Cs films, and the control were treated fruits. Packaged fruits were left at room temperature for 9 days to evaluate the shelf life and quality.

**Fig. 12:**
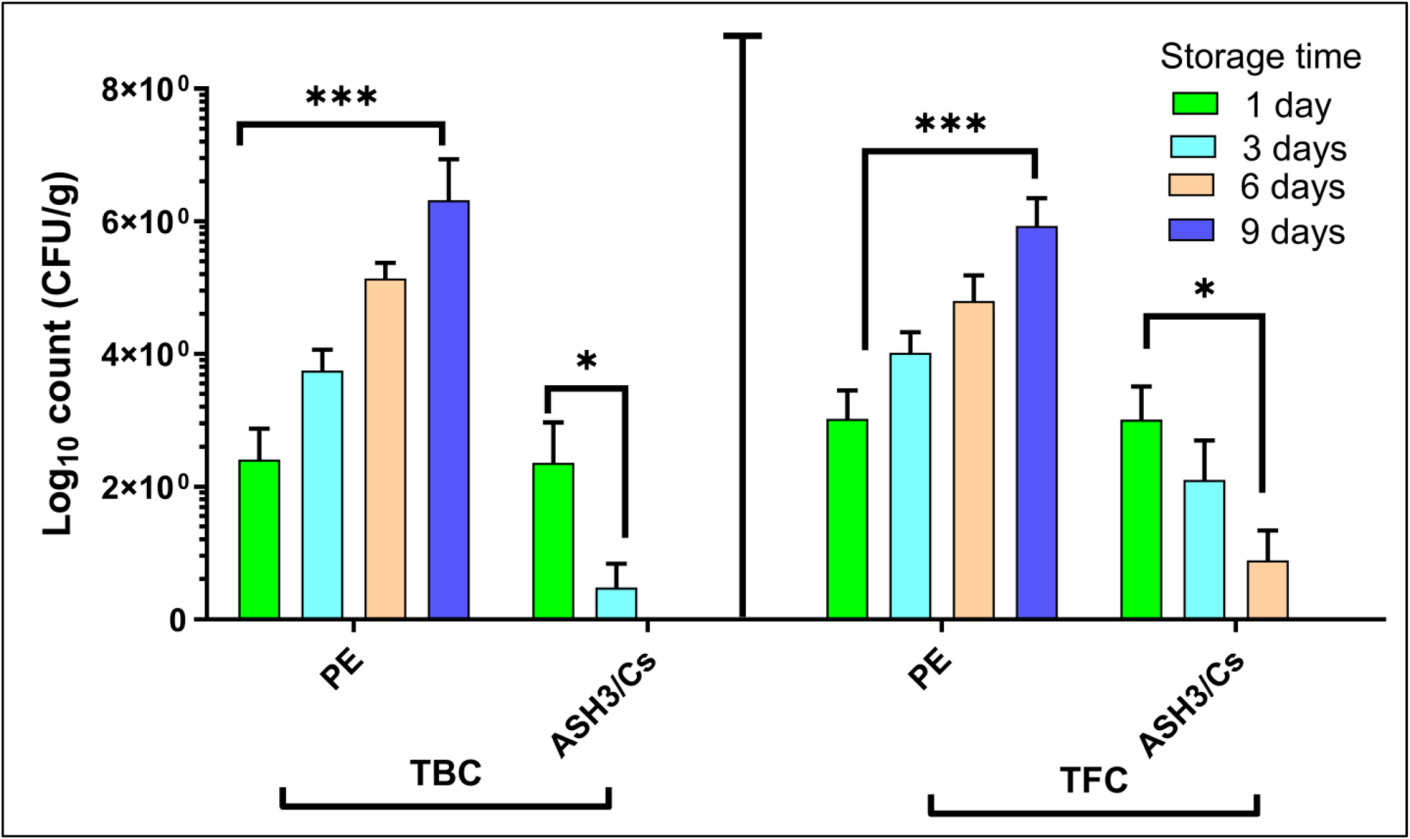
Total bacterial and fungal counts (TBC and TFC) of fresh-cut strawberries during the storage period (9 days).

The information in Fig. 12 showed how the microbiological profiles of the traditional polyethylene (PE) films and the ASH3/CS films change under various storage circumstances. This significant difference in the overall microbial counts between the two materials is an important finding. Throughout the 1, 3, 6, and 9-day storage periods, the PE films exhibited considerably greater TBC and TFC than the ASH3/CS film. The TBC and TFC in the PE film samples showed a more marked increase with increasing storage duration, suggesting a quicker growth rate of bacterial populations. On the other hand, the microbiological profile of the ASH3/CS films was much more consistent and under control, with comparatively lower TBC and TFC values sustained throughout the 9-day storage period. The findings indicated that the ASH3/CS film exhibits higher antibacterial activities. This may be due to the inclusion of ASH extract into the Cs matrix. The ASH extract's antibacterial properties probably prevent bacterial and fungal microbes from growing, which explains why there were less microorganisms in the ASH3/CS film than in the PE films. The ASH3/CS film's superior ability to retain the samples' microbiological quality for a more extended period of storage points to their potential benefits in applications like food packaging, where microbial growth control is essential to preserving product safety and shelf life. According to the results, the ASH3/CS films could work better in these kinds of applications than traditional PE films.

**Fig. 13:**
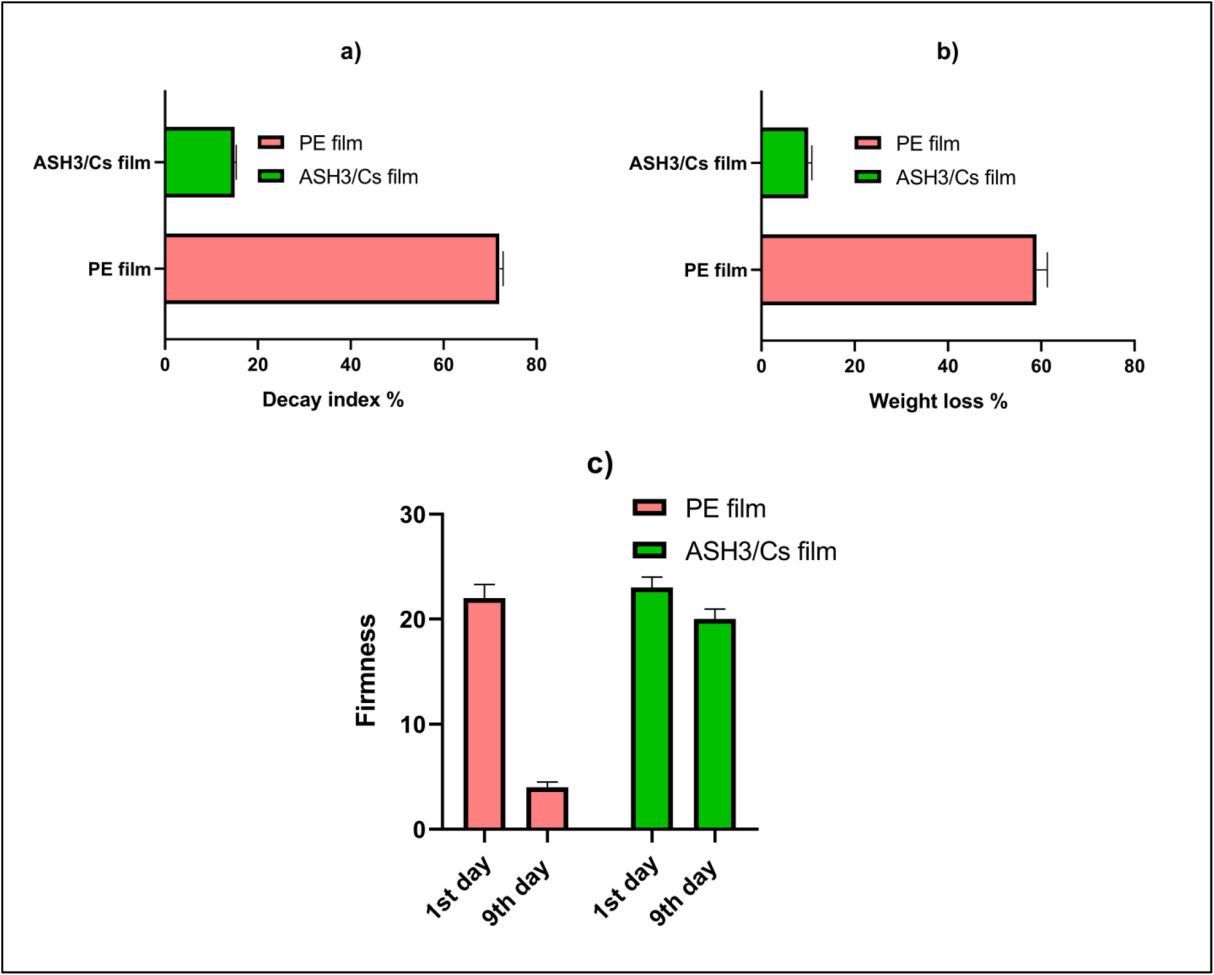
Quality of fresh-cut strawberries during storage period (9 days), (a) decay index, (b) weight loss; and (c) firmness.

Pieces of fresh-cut strawberries were studied for quality features after being stored for 9 days based on weight loss, decomposition index, and hardness. The degradation index continued increasing and dropping by the storage terminal. On the contrary, ASH3/Cs film delayed the breakdown rate in the strawberries fruits as observed over a 9-day storage period. The rate of decline of fruits took a different shape in those handled with PE materials than in those packed with ASH3/Cs film (Fig. 13(a)). Moreover, PE revealed an elevated degradation rate after the storage duration (9 days). Strawberries wrapped with PE material exhibited more quality decline after the storage period compared to those packaged with ASH3/Cs film. Regarding, weight loss and dry matter consumption caused by respiration led to a noticeable trend of fresh-cut strawberries weights in each group declining throughout the 9-day storage period, as illustrated in Fig. 13(b) This drop was less severe in the ASH3/Cs film group than in the PE-packaged group. In the present study, over the period of storage, the hardness of fresh-cut strawberries fruits was reduced progressively in all the samples studied, and this phenomenon is represented in Fig. 13(c). During the first day, all fruits maintained similar firmness. However, after 9 days of storage, the fresh-cut strawberries fruit packaged with the ASH3/Cs film recorded considerably greater firmness than the PE material. The results illustrated in Fig. 13 clearly demonstrate that ASH3/Cs films significantly improve the quality of fresh-cut strawberries during storage by reducing decay, minimizing weight loss, and maintaining firmness. These results highlight the potential of ASH3/Cs films as an effective food packaging material, capable of extending the shelf life of perishable fruits and enhancing their overall quality.

**Fig. 14:**
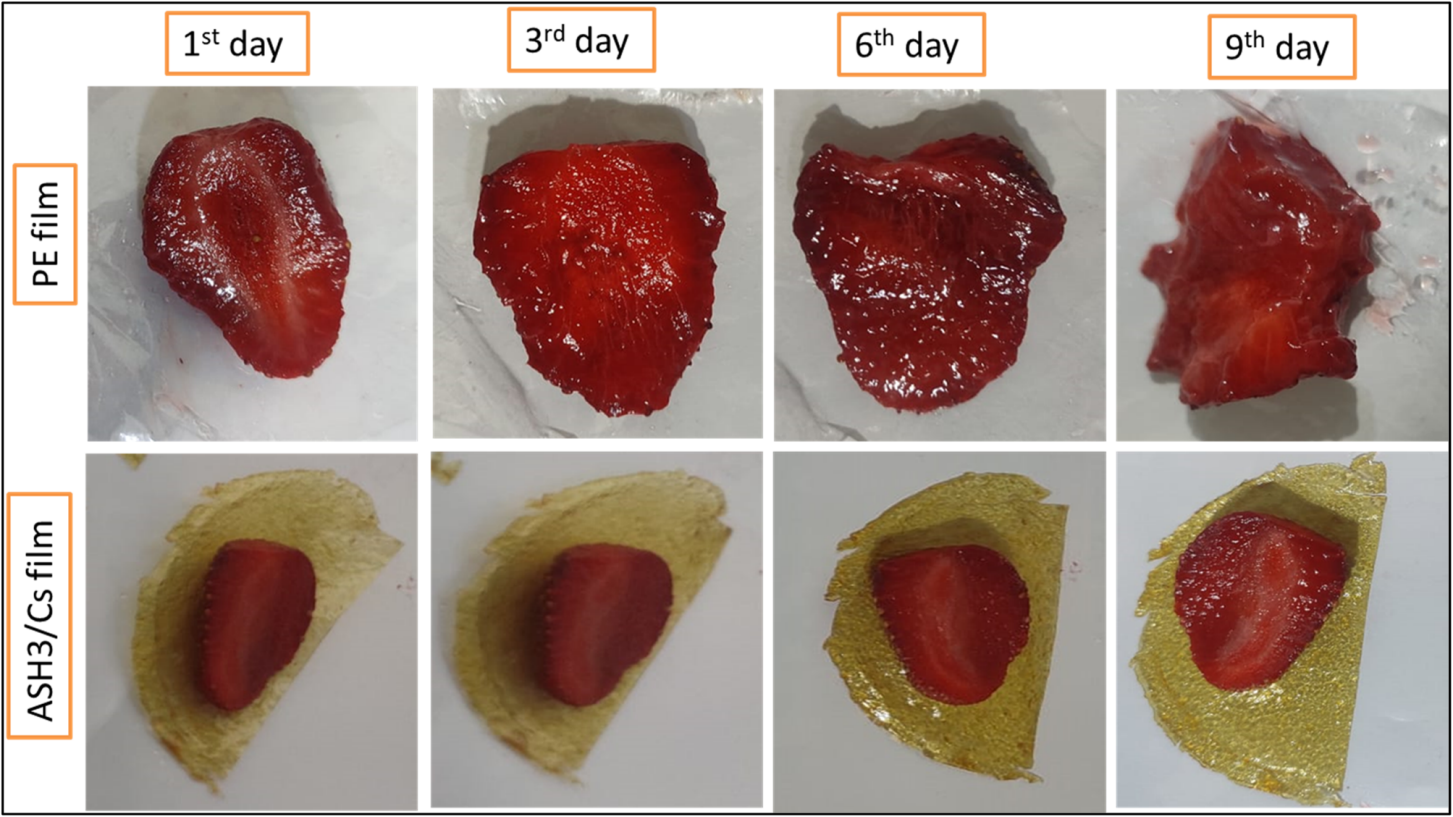
Visual inspection of fresh-cut strawberries after wrapping with PE film and ASH3/Cs films at 1, 3, 6, and 9 day of storage at 25 °C and 65% of humidity.

The investigation used visual inspection to capture snapshots of freshly cut strawberries at four distinct intervals (1, 3, 6, and 9 days) to assess the extent to which the ASH3/Cs film preserved the quality of the fruit in comparison to polyethylene (PE) material. The photographs showed that during the observation period, the color and hardness of the strawberries included in the ASH3/Cs film were not significantly different (Fig. 14). On the other hand, fresh-cut strawberries from the second group (PE film) showed noticeable changes in texture and color. The PE-coated strawberries preserved their soft texture and light color, confirming the film's efficacy as a protective barrier. Moreover, the strawberries enveloped in PE film exhibited noticeable color alterations and moisture depletion. The testing photographs demonstrated that the ASH3/Cs film effectively preserved the strawberry's physical and visual attributes. The protective barrier provided by the ASH3/Cs coating increased the fruit's shelf life, which reduced moisture loss and color deterioration. This implies that the ASH3/Cs film may effectively preserve perishable fruit quality while it is transported and stored.

The study on conductive cellulose nanofiber (CNFene)-reinforced PLA highlights how nanomaterials can improve mechanical strength, crystallization, and thermal stability, a concept relevant to our incorporation of ASH extract in Cs films.^69^ While CNFene enhances PLA's crystallinity and thermal properties, ASH extract enhanced antimicrobial and antioxidant functionalities to Cs films, extending the shelf life of fresh-cut strawberries. In another study, the curcumin-incorporated biocomposite films demonstrated their effectiveness as secondary packaging for cut potatoes, showcasing their potential for active and sustainable food packaging applications.^70^

## Conclusion

The blending of ASH extract with chitosan (Cs) leads to the formation of biodegradable active materials. The incorporation of ASH changed the morphological surface of Cs film. The powerful antioxidant properties of ASH extract contribute to reducing oxidative stress and spoiling, which in turn improves the preservation of food. The ability of ASH extract to fight microbes is due to various mechanisms, such as disrupting cell membranes, inhibiting protein synthesis, and enzyme inhibition. In the food industry, ASH extract can be used in edible coatings, active packaging materials, and natural preservatives to protect against microbial contamination during storage and transport. The study emphasizes the potential of ASH3/Cs films as effective food packaging material. Results concluded that Cs-based food packaging materials could be highly innovative by incorporating extracts derived from agricultural waste rich in polyphenols, such as date kernels, pomegranate peels, or grape pomace. These natural additives not only enhance the antioxidant and antimicrobial properties of the packaging but also provide a sustainable approach to utilizing agricultural by-products. Polyphenol-rich extracts could effectively extend the shelf life of fresh produce by minimizing microbial growth and oxidative spoilage, aligning with eco-friendly, biodegradable, and waste-reducing packaging solutions.

## Data availability

All data generated or analyzed during this study are included in this published article.

## Author contributions

Mohamed Gouda: conceptualization, supervision, investigation, methodology, resources, formal analysis, data curation, funding acquisition, writing – original draft, writing – review & editing. Hany M. Abd El-Lateef: conceptualization, investigation, methodology, resources, formal analysis, data curation, funding acquisition, writing – original draft, writing – review & editing. Nashi K. Alqahtani: investigation, methodology, writing – original draft, writing – review & editing. and Manal F. Abou Taleb: methodology, formal analysis, writing – original draft, writing – review & editing. Ibtisam Alali: methodology, formal analysis, writing – original draft, writing – review & editing.

## Conflicts of interest

The authors declare that they have no competing interests.
